# Feasibility study of sustained release dosage forms for incrementally modified drug by domestics pharmaceutical industry in Thailand

**DOI:** 10.12688/f1000research.142745.1

**Published:** 2023-11-27

**Authors:** Charkkrit Hongthong, Rungpetch Sakulbumrungsil, Khunjira Udomaksorn, Sitanun Poonpolsub, Osot Nerapusee, Manthana Laichapis, Nusaraporn Kessomboon

**Affiliations:** 1 Faculty of Pharmaceutical Sciences, Khon Kaen University, Nai Mueang, Khon Kaen, 40002, Thailand; 2 Department of Social and Administrative Pharmacy, Faculty of Pharmaceutical Sciences, Chulalongkorn University, Bangkok, Bangkok, 10330, Thailand; 3 Department of Pharmacy Administration, Faculty of Pharmaceutical Sciences, Prince of Songkla University, Hat Yai, Songkhla, 90110, Thailand; 4 The Office of International Affairs on Health Consumer Protection, Food and Drug Administration Thailand, Nonthaburi, Nonthaburi, 11000, Thailand; 5 Division of Social and Administrative Pharmacy, Faculty of Pharmaceutical Sciences, Khon Kaen University, Nai Mueang, Khon Kaen, 40002, Thailand

**Keywords:** feasibility study, sustained release, incrementally modified drug, pharmaceutical industry in Thailand

## Abstract

**Background:**

Incrementally modified drugs (IMDs) are anticipated to improve drug efficacy and compliance. Compared to new chemical entities, the research and development costs for IMDs are lower. The domestics industry currently focuses on manufacturing both generic and new generic drugs. To promote the growth of the industry, it is crucial to investigate the feasibility of developing IMDs.

**Methods:**

This necessitates evaluating all five aspects, including needs, market, scientific, technological, and regulatory feasibility from January to December 2022. A mixed methods approach, combining literature review and in-depth interviews conducted online via Zoom. Qualitative data analysis will be performed through thematic analysis.

**Results:**

Needs: medical needs, six out of seven physicians expressed a preference for sustained release (SR). The domestics industry is actively engaged in the research and development of SR as well. Market feasibility: The compound annual growth rate for SR in 2019 was found to be 3.97 percent, SR encompassing all nine of the 14-drug group. Scientific feasibility is not a concern as the industry has the capability to develop drugs. Technological feasibility: The IMDs technology readiness level is at level 4 (high level). The manufacturing of SR utilizes a matrix technique, membrane system, and pellets. Regulatory feasibility: Although have registration guidelines for IMDs, no industry has successfully registered IMDs yet.

**Conclusions:**

SR has demonstrated feasibility for IMDs development in all aspects except for the challenging issue of drug registration.

## Introduction

Thailand’s new drug registration process adheres to ASEAN Harmonization guidelines since September, 2007 and contain 7 new drugs as follows: 1) new chemical entities (NCEs) 2) new indication 3) new combination 4) new delivery system 5) new route of administration 6) new dosage form 7) new strength. Incrementally modified drugs (IMDs) according to the Thai FDA ^1^ definition is a new drug type of drug that is the original chemical drug from no. (2) - (7). IMDs aim to enhance patient convenience, compliance, and effective treatment of diseases. The definition provided by Lapteva (2015) for the United States of America (USA) is the development of pharmaceutical products using the original active pharmaceutical ingredients (API). ^2^ IMDs aim to improve drug efficacy, minimize side effects, lower the risk of drug interactions, and enhance drug compliance. The Ministry of Food and Drug Safety (MFDS) of South Korea defines IMDs as drugs that have the same active ingredient as the original drug but with modified properties or drug types aimed at improving their efficiency. ^3^

The number of newly registered drugs in the USA has decreased, while the expenditure on research and development has continued to increase, indicating a crisis in the domestic pharmaceutical industry. However, IMDs have been on the rise since the 1970s. ^4^ This could be due to the following reasons: 1) research and development costs are less than NCEs, with the cost of research and development being used, NCEs use 10 times more than IMDs. 2) The USFDA has begun approving more registrations of IMDs since the 1970s. 3) There is a higher prescription rate for IMDs. 4) Within five years of USFDA approval, it was found that as many as 51 percent of IMDs were ordered, while NCDs have been just 17 percent over the same period. 5) IMDs can increase the patient’s drug access who has had previous experience with the drug. And 6) IMDs take a shorter time for drug registrations than NCDs (IMDs take 5-6 years, NCDs 11 – 12 years) and have fewer monopolies.

IMDs in South Korea are gaining a competitive edge in the drug market compared to new generics due to their ability to reduce costs and shorten the research and development time of new drugs. The South Korean government has a policy of supporting and promoting the domestic pharmaceutical industry specifically for the development of IMDs. As a result, the South Korean domestic pharmaceutical industry has registered 29 new chemical entities (NCEs) and 82 IMDs from 2000 to the present. The compound annual growth rate (CAGR) was used to measure the growth rate of the pharmaceutical market. It was found that the number of pharmaceutical products had a CAGR of 4.6 percent, while exports of pharmaceutical products had a CAGR of 11.5 percent and drug imports had a CAGR of 2.8 percent. The size of the pharmaceutical market had a CAGR of 3.1 percent. ^5^

The development of IMDs in India brings added value and benefits to the domestic pharmaceutical industry. India has been a leader in manufacturing new generic drugs, but with the intellectual patent (IP) protection of prototypes, the industry has shifted focus towards the research and development of IMDs to enhance efficacy, safety, and compliance. ^6^ During the period from 1990 to 2000, the domestic pharmaceutical industry in India saw a 65 percent increase in IMDs registrations, highlighting the value and benefits of IMDs to the industry. These benefits can be summarized as follows: 1) IMDs can improve drug stability in India’s tropical climate. 2) IMDs can enhance the treatment of pandemic diseases in India by developing new dosage forms, changing the route of administration, and creating combination drugs that can stop the spread of disease. 3) IMDs encourage continual research and development in the domestic pharmaceutical industry, as the IMD market is projected to reach a value of 20 billion dollar by 2015. 4) IMDs result in lower treatment costs as they are carefully selected for the treatment of patients in India. And 5) IMDs increase patient access and drug market competition, leading to more affordable drug prices.

It was found that there are 163 pharmaceutical industries in Thailand at present, however, only 144 of them have been granted the GMP PIC/S certification as of February 2020. ^1^ During the period of 2013-2015, it was observed that the domestic pharmaceutical industry in Thailand experienced a decline of 11.65 percent. The size of the industry during this period showed that the majority (80 percent) of the industry had a value of less than 500 million Baht, while only 20 percent of the industry had the potential to innovate and compete at a regional level. In terms of pharmaceutical exports, Thailand only accounts for 10 percent of the total production of medicines in Southeast Asia, including Cambodia, Laos, Myanmar, and Vietnam. ^7^

Most of the domestic pharmaceutical industry focuses on producing generic drugs and new generic drugs. Research and development trends in the industry involve working on prototypes that have expired patents and improving their properties, such as enhancing drug stability and solubility. Over the last decade, pharmaceutical firms have established dedicated research and development departments and made significant investments in this area. Furthermore, they have employed new technologies to expand the range of pharmaceutical products they develop. ^1^ There has been little progress in new drug research and development, as evidenced by the low number of registrations approved by the Thai FDA. Only GPOVIR has been approved in new combination, which may be due to the domestic pharmaceutical industry’s shortcomings. There is a lack of relevant agencies for new drug registration.

A feasibility study (FS) aims to identify possible pathways for a project, considering multiple approaches that can address the system’s problem with minimal waste of time and cost. ^8^ Moreover, the results are deemed satisfactory in assisting executives to make data-driven decisions with ease. ^9^ Most FS can be classified into five categories based on the TELOS framework, which includes technical, economic, legal, operational, and schedule considerations. ^10^^–^^12^ Hence, this FS aims to evaluate the feasibility of the domestic pharmaceutical industry’s development of a dosage form for IMDs. It examines various factors that influence the potential outcomes, such as technical, marketing, regulatory, scientific, and needs, to assist decision-makers in determining the appropriate course of action. Regarding the examination of financial feasibility conducted in Manthana’s thesis, ^13^ the study encompasses an exploration of cost frameworks and the evaluation of expenses associated with investments in the manufacturing sector of IMDs. Additionally, the thesis incorporates an analysis of the financial feasibility of IMDs, incorporating sensitivity analysis and scenario analysis.

To support the development policy of the domestic pharmaceutical industry along the value chain, it is essential to conduct research and develop IMDs using a high-technology platform for manufacturing. This would reduce drug imports and improve patient access to drugs, promoting sustainable self-reliance in the domestic pharmaceutical industry. Hence, this research aims to examine the feasibility of sustained-release dosage form development for IMDs by the domestic pharmaceutical industry. The study evaluates all five feasibilities of developing IMDs, including scientific, marketing, needs, technology, and regulatory aspects.

## Methods

A Mixed-methods research will be conducted from January to December 2022. We were hired to assess the feasibility of sustain released dosage forms development by the domestic pharmaceutical industry. The study collected data from literature review and structured in-depth interviews. Conduct semi-structured, in-depth interviews with participants using a snowball and purposive sampling approach until data saturation is achieved. Utilize Zoom for online interviews lasting approximately 30-60 minutes and record audio for subsequent data analysis. It is important to note that the audio recordings will be securely destroyed at the conclusion of the study.

The Human Research Ethics Committee of Khon Kaen University reviewed this study (HE642276) approval January 20, 2022. Each participant signed an informed consent form by mail after receiving a comprehensive explanation of the study’s objectives. Transcription of the interviews ^14^ maintained the anonymity and confidentiality of information through the use of code numbers, with exclusive access granted only to the researchers.

The feasibility assessment in this study is adapted from Thomayant et al.’s research. ^15^ Factors affecting feasibility covering 5 domains: including needs, market, scientific, technological, and regulatory were assessed. The study was then be divided into 5 parts as follows.

**Part 1:**Needs analysis was performed on 2 perspectives:

1.1 Medical needs: Engaging in extensive interviews with 5 expert physicians representing diverse medical domains or until data saturation, snowball selection criteria for experts include individuals with the capability to assess the necessity for the development of IMDs, specializing in dosage forms, or those with expertise in medical or pharmaceutical professions. Employ the following set of questions during the interview: Is there a clinical necessity for sustained release dosage forms?

1.2 Domestic pharmaceutical industry needs: Conducted interviews with purposive sampling five domestic pharmaceutical industry possessing the capability to develop IMDs or until data saturation. Utilize the ensuing set of questions throughout the interview: Is there a demand within the domestic pharmaceutical industry to produce this dosage forms?

**Part 2:** Market feasibility, incorporating an assessment of the marketing market stance, this phase encompassed the data analyzed from the drug database report of the Thai FDA and in-depth interviews with five experts well-versed in domestic pharmaceutical marketing or until data saturation. Inclusion criteria involve experts with proficiency in the marketing of SR dosage forms. These endeavors aimed to ascertain the present market positioning and pivotal distinguishing factors.

**Part 3:** Scientific feasibility was determined by examining relevant scientific literature for each dosage form. The decision to develop SR dosage forms was based on the active ingredient’s half-life. The investigation involved the selection of expert-validated keywords, specifically "half-life" followed by a search conducted in the Scopus, and Google Scholar databases.

**Part 4:** Technological feasibility, the conducted interviews necessitated the involvement of purposive sampling 5 individuals with substantial experience and expertise in the domain of domestic pharmaceutical manufacturing, focusing on the subsequent aspects: 4.1 IMDs Technology readiness level (IMD-TRLs) including the following:

4.1.1 Basic research (scientific review of reference drug (knowledge base), feasibility study and risk management, and preclinical studies for proof of concept.

4.1.2 Laboratory prototype (demonstration of proof of preclinical studies, vivo models, pharmacokinetics, safety, and toxicity of drug formulations, pharmacokinetics, and pharmacodynamics of drug formulations under GLP, begin of GMP product development, and prepare a protocol of Pre- Investigational New Drug (IND) application and consult Thai FDA.)

4.1.3 Prototype development (production of a pilot lot under GMP, beginning of phase 1 clinical trials in a small number of humans and submission of investigational new drug (IND) application, phase 2 clinical trials for preliminary evidence, collection of safety, toxicity, and immunogenicity data, submission of updated IND application, and phase 3 clinical trial for risk-benefit assessment and preparation of New Drug Application (NDA) for submission to regulatory authority.)

4.1.4 Production (submission of New Drug Application (NDA) and Thai FDA approve and launching and marketing of new pharmaceutical product and post-marketing surveillance.)

4.2 Operational/Manufacturing feasibility

**Part 5:** Regulatory feasibility, the in-depth interviews encompassed the insights and expertise of five individuals, including professionals in regulatory affairs, representatives from the Thai FDA, and academic experts. These discussions centered around topics such as regulatory pathways and the requisite quantity and nature of studies mandated.

### Data analysis

The drug database report spanning from 2010 to 2019 from the Thai FDA was analyzed using Excel. Descriptive statistics were presented as percentages.

Concerning qualitative data, interview transcripts ^14^ were generated, and manual coding was employed to investigate key themes through manual thematic analysis.

## Results

The total value of drugs in Thailand has been 224,831.93 million Baht since 2019, with domestic production accounting for 67,963.56 million Baht (30.23 percent) and imported drugs accounting for 148,031.43 million Baht or 65.84 percent. The pharmaceutical manufacturing industry mainly produces generic drugs and new generic drugs (95.50 percent). A feasibility study was conducted on SR dosage forms in five areas, needs, market, legal, scientific, and technological aspects.

### Part 1: Needs analysis

**Figure 1.**
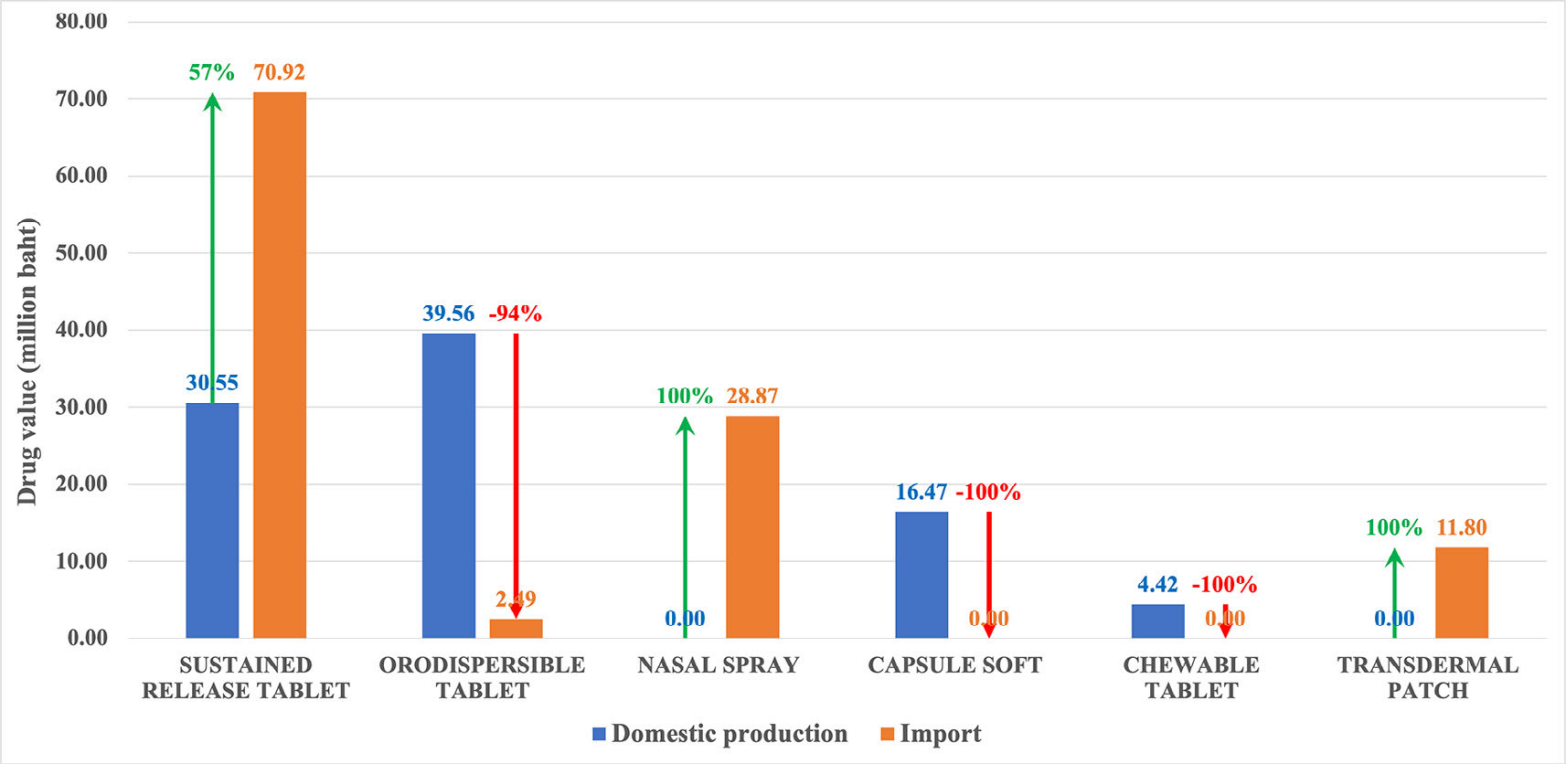
2019 pharmaceutical imports - dosage forms of new generic drugs in Thailand.

Figure 1. indicates that most pharmaceutical dosage forms that demand advanced manufacturing technology are imported and have high value, including SR, nasal spray, and trans-dermal patch dosage forms.

The delayed-release dosage form was identified as having a compound annual growth rate (CAGR) of 187.54 percent, while the orodispersible tablet had a CAGR of 14.86 percent, making them potential dosage forms for the development of IMDs by the domestic pharmaceutical manufacturing industry, CAGR of transdermal patch, chewable tablet, sustained release, nasal spray, powder, and soft gelatin capsule. (14.13, 7.85, 0.18, -0.45, -1.95, and -6.3 percent respectively) (Database of the year 2011 – 2019 from Thai FDA.)

According to the Thai FDA’s definition, a new delivery system refers to a drug that has a novel dosing format. This involves the creation of a fresh drug delivery mechanism that causes significant changes in the drug’s pharmacokinetics compared to the original version. ^1^

SR is a type of medication formulation designed to reduce dosing frequency and extend drug absorption over an extended period. This formulation can improve medication compliance, particularly among patient groups with poor compliance, such as the elderly, those on multiple medications, and individuals with chronic diseases. In 2019, the total value of SR dosage forms was 5,448.25 million Baht, comprising new drugs, generic drugs, and new generics valued at 3,426.21 million Baht, 1,561.40 million Baht, and 101.47 million Baht, respectively. The value of imports, production, and packaging for SR dosage forms was 5,282.11 million Baht, 63.20 million Baht, and 102.94 million Baht, respectively. SR dosage forms have exhibited a Compound Annual Growth Rate (CAGR) of 3.97percent.

Following are the outcomes obtained from the in-depth interviews conducted to identify the medical and developmental requirements of the pharmaceutical industry:

1. Medical needs: after conducting in-depth interviews, it came to light that six of the interviewed physicians emphasized the requirement for drug development utilizing SR dosage forms. The objective was to improve patient compliance and reduce the frequency of dosing.
2. Needs of the domestic pharmaceutical industry: the expertise provided insight into a noticeable demand for SR development, with most of these companies presently immersed in research and development endeavors within this domain.

### Part 2: Marketing feasibility

In Thailand, the drug market is dominated by government hospitals, private hospitals, and drug stores, accounting for 60, 25, and 15 percent of the buyers, respectively. Government hospitals are the major buyers, and the Government Pharmaceutical Organization (GPO) holds a monopoly as the primary seller. Most of the domestic pharmaceutical manufacturing industry focuses on producing generic and new generic drugs for the local market.

Upon analyzing the global market, it was observed that the Compound Annual Growth Rate (CAGR) of the SR dosage forms market was 6.6 percent. However, in the Thailand local market, the CAGR of SR dosage forms from 2011 to 2019 was found to be 3.97 percent. The drug market in Thailand is mostly dependent on imports, with imports accounting for more than 80 percent of the market. In the case of the SR dosage forms market, the value of production and import in 2019 was 5,345.31 million Baht, which exceeded the value of domestic production and constituted 97.63 percent of the value of imports in comparison to domestic production.

Based on an analysis of the Thai FDA database for the year 2019, it was observed that SR dosage forms were a dosage forms that covered all nine out of 14 Anatomical Therapeutic Chemical (ATC) groups at level 1, as listed below:

1. A (Alimentary tract and metabolism)
2. B (Blood and blood-forming organs)
3. C (Cardiovascular system)
4. G (Genito urinary system and sex hormones)
5. J (Ant-infectives for systemic use)
6. L (Antineoplastic and immunomodulating agents)
7. M (Musculoskeletal system)
8. N (Nervous system)
9. R (Respiratory system)

**Figure 2.**
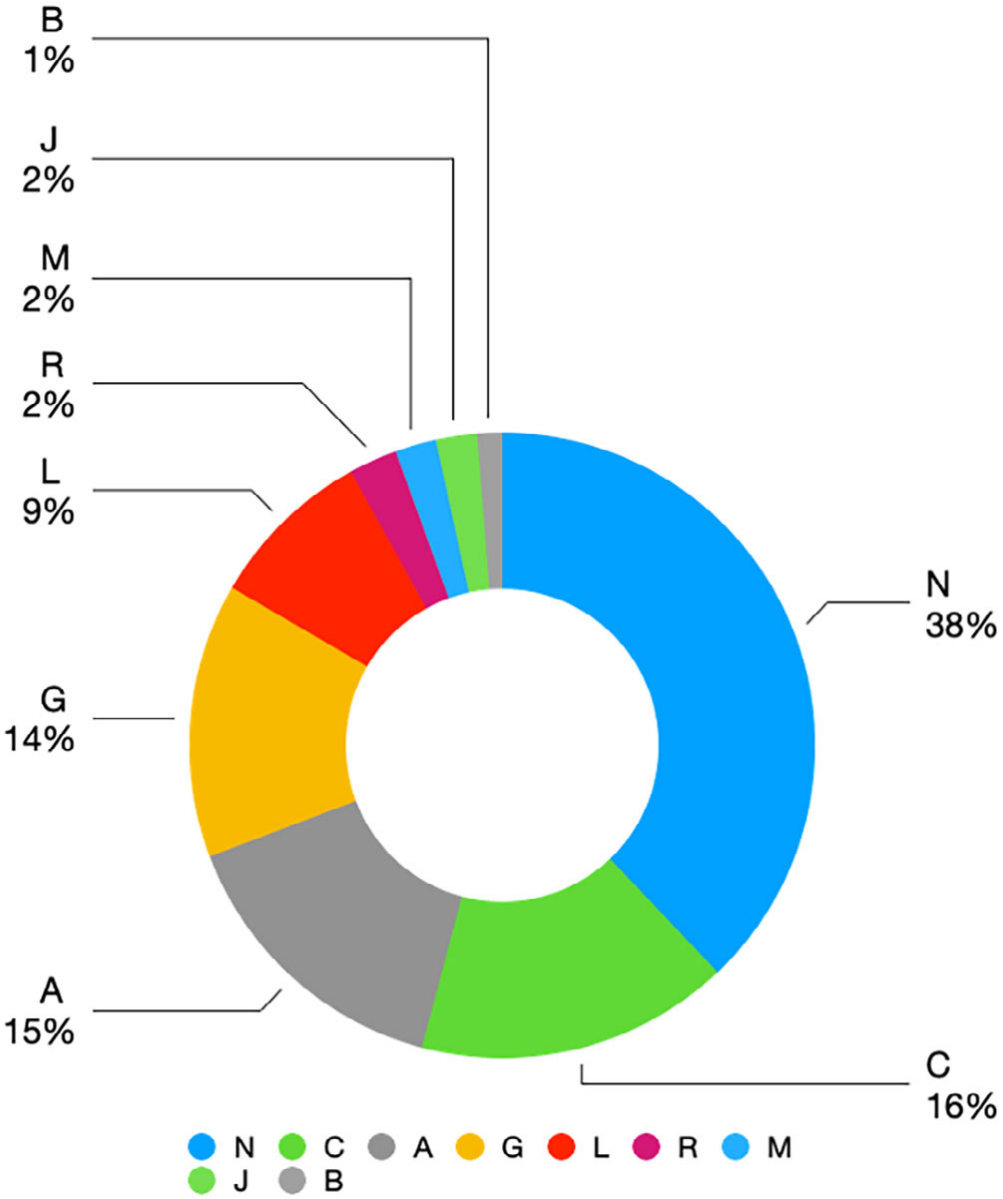
Value drug (billion baht) and percentage of level 1 ATC classification of SR dosage form. Value drug (billion baht) and percentage of level 1 ATC classification of SR dosage form.

The N group was the most prevalent ATC level group, accounting for 38 percent of the market, followed by the C group, A group, and G group at 16, 15, and 14 percent, respectively. There are only three groups of drug production and registration in Thailand, namely the A group, N group, and C group, which are divided among four domestic producers and nine importers. A comparison of prices revealed that domestically produced 1,000 mg blood glucose-lowering SR tablets cost 350 Baht per unit, while imported ones cost 240 Baht per unit for the SR dosage forms. It is anticipated that the number of drug registrations for SR dosage forms in Thailand will remain stable, as shown in Figure 2.

### Part 3: Scientific feasibility

To assess the scientific feasibility of developing IMDs for SR dosage forms, a search was conducted for the physiochemical properties of drugs within the ATC level 3 groups. These groups were selected from the top 10 highest-value groups in Thailand. The development of an SR dosage forms drug involves considering various physiochemical and pharmacokinetic properties of the drug. The following parameters should be taken into consideration ^16^^,^^17^:

1. molecular size less than 600 Dalton’s
2. aqueous solubility more than 0.1 mg/ml
3. partition coefficient $k_{o/w}$ 1-2
4. dissociation constant $P_{\mathrm{ka}}$ (acidic drugs, $P_{\mathrm{ka}}$ >2.5 and basic drugs, $P_{\mathrm{ka}}$ <11)
5. elimination half-life between 2-6 hrs.

Experts have stated that a critical factor in the decision to develop SR dosage forms is the active ingredient’s half-life, which should fall between 2-6 hours. Out of the 10 treatment groups analyzed, it was found that 9 had physiochemical properties suitable for development into SR dosage forms. From a list of 138 drugs, 40 were identified as having potential for SR dosage forms. In the United States, 26 drugs were developed into SR dosage forms, but only 8 of them had eligible half-lives between 2 and 6 hours. In Thailand, there are currently only 3 drugs produced as SR dosage forms: felodipine, gliclazide, and metformin. (Please note that this list of 3 drugs is not exhaustive.)

The following drugs with half-life properties between 2-6 hours have already been developed into SR dosage forms (see Table 1).

**Table 1.** Possible drugs to develop as an SR dosage forms have been compiled in a list.

No.	ATC level 3 groups	Possible drugs
1.	A02B (Drugs for peptic ulcer and gastro-esophageal reflux disease (GERD)) (3/14)	Ranitidine, Famotidine, Omeprazole
2.	C08C (Selective calcium channel blockers with mainly vascular effects) (1/8)	Nifedipine
3.	A10B (Blood glucose lowering drugs, excl. Insulins) (6/24)	Glymidine, Acarbose, Miglitol, Voglibose, Rosiglitazone, Vildagliptin
4.	M01A (Anti-inflammatory and antirheumatic products, non-steroids) (8/21)	Indomethacin, KetorolacI, buprofen, Fenoprofen, Mefenamic, Meclofenamic, Lumiracoxib, Nimesulide
5.	J01C (Beta-lactam antibacterial, Penicillin) (4/5)	Amoxicillin, Piperacillin, Sulbactam, Tazobactam
6.	N03A (Antiepileptics) (2/12)	Oxcarbazepine, Gabapentin
7.	R06A (Antihistamines for systemic use) (3/13)	Dimenhydrinate, Levocetirizine, Triprolidine
8.	N05A (Antipsychotics) (2/19)	Trifluoperazine, Chlorpromazine
9.	C07A (Beta blocking agents) (2/9)	Atenolol, Labetalol

### Part 4: Technological feasibility

**Technology readiness feasibility by IMDs technology readiness level (IMD-TRLs)**

Based on the in-depth interviews, ^20^ it was found that the top 5 domestic pharmaceutical industries are prepared to develop IMDs at the 4th level, as they have already conducted research and development of new generic drugs in the SR dosage forms. The process for obtaining approval for registration with the Thai FDA is as follows: All industries have a basic level of readiness, while only one industry has achieved stage 4 readiness. However, it should be noted that this industry has developed a new generic drug in SR dosage forms.

**Operational/Manufacturing feasibility**

Based on the in-depth interviews, it was found that the top 5 domestic pharmaceutical industries have the necessary operational and manufacturing expertise and analytical processes to formulate SR dosage forms using the matrix technique. The manufacturing process, membrane systems, and pellet technologies are simple and can be researched and developed without the need for significant investments in new equipment. Additionally, these processes are widely used in the industry. Due to the need for specialized machinery and high investment, the domestic pharmaceutical industry is not capable of producing SR dosage forms using the osmotic pump technique.

**Quality control**

Based on the interviews of 5 domestic pharmaceutical industries, it was found that most of the domestic pharmaceutical industries have access to analytical tools and possess the capability to perform analysis of SR dosage forms within their own facilities. Four out of five pharmaceutical industries have the capability to produce SR dosage forms using matrix technique and membrane systems methods, according to the interviews conducted. Analytical processes, all industries can perform dissolution and stability analysis for their own SR dosage forms.

### Part 5: Regulatory feasibility

**Registrational feasibility/Regulatory pathway and number/type of studies required.**

New drug registration (ASEAN HARMONIZATION) Guidelines ^18^ ASEAN common technical dossier (ACTD) for the registration of pharmaceuticals for human use in new drug consists of 4 parts as follows:

Part 1: Administration data and product information

Part 2: Quality document

Part 3: Safety: non-clinical document

Part 4: Efficacy: clinical document

The technical dossier required for the registration of IMDs includes non-clinical and clinical studies. This is applicable to 6 types of new developments, including new indications, new combinations, new drug delivery systems, new routes of administration, new dosage forms, and new strengths. The licensee can refer to the guidelines and recommendations provided by the Thai FDA for conducting non-clinical and clinical studies. These recommendations and guidelines were released on February 20, 2019, for the registration of new drug developments based on previously approved chemical drugs. ^19^

If there is a change in the drug delivery system, non-clinical studies such as pharmacology, pharmacokinetics, and toxicology need to be conducted for the development of IMDs. Clinical studies are also required. Bioequivalence studies shall be conducted to demonstrate that the experimental drug exhibits the same duration of release as the reference drug without the release of dose dumping. Pharmacokinetic parameters were consistent with the single dose, two-period, two-treatment, two-sequence crossover study (feed and fed conditions). Except in cases where the product tends to accumulate accumulation, a multiple-dose, two-period, two-treatment, two-sequence crossover study (feed and fed conditions) was required.

Regulatory pathway in Thailand, Thai FDA issued an announcement entitled “Preparation before submission of drug formulary registration for new drug product development” as a procedure and process for applying for consultation. For the industry that wants to request approval for the registration of IMDs on February 6, 2020. And on January 19, 2022, the Thai FDA issued an announcement entitled “Guidelines for the preparation of registration documents and preparation before submission of drug formulary registration of new drugs developed from chemical drugs”. For the industry that wants to apply for IMDs. The regulatory guidelines are clear, but no industry has been able to successfully register IMDs. This may be due to the process and personnel having to develop the ability to register IMDs.

During interviews with the pharmaceutical industry, which has the capability to research and develop new drug delivery dosage forms, the following issues were identified: The non-clinical and clinical study recommendations and more specific guidance. The consultation process does not match the announcement, leading to insufficient assistance provided to the applicant and the registrar to effectively collaborate in registering the drug.

Summary for feasibility study (see Table 2).

**Table 2.** Summary for Feasibility Study.

Feasibility Study	Study topic	Result
Needs analysis	1. medical needs	SR to enhance patient compliance and minimize dosing frequency
	2. industry needs	most of these companies are currently engaged in research and development activities in SR
Marketing feasibility	1. CAGR (2011 to 2019)	3.97 percent
	2. covers ATC group	total 9 groups (N - Nervous system 38 percent)
Scientific feasibility	1. half-life 2-6 hrs	Nine out of ten groups have identified 31 potential drugs for development
Technical feasibility	1. IMD-TRLs	Basic research (4) and Production (1)
	2. production capability	matrix technique, membrane systems, and pellet technologies
	3. quality control	dissolution and stability analysis
Regulatory feasibility	1. regulatory pathway	Thai FAD announces wide guideline
	2. ability to approve registration	The consultation process does not match the announcement

## Discussion and suggestions

Most FS can be classified into five categories based on the TELOS framework follows ^10^^–^^12^:

1. Technical feasibility: The assessment includes evaluating the readiness of the structure for operations, assessing the operational capabilities of the action plan, and ensuring sustainability in the operations.
2. Economic feasibility: This involves conducting a cost-benefit analysis which includes evaluating the net present value (NPV), breakeven point, internal rate of return (IRR), payback period (PB), and other relevant financial metrics.
3. Legal feasibility: The objective is to ensure that the project adheres to legal, ethical, patent, and regulatory requirements, as well as obtaining necessary permits, such as conducting an environmental impact assessment (EIA).
4. Operational feasibility: This involves a study of the necessary requirements for the project’s compatibility, including organizational culture, policy systems, and stakeholders involved in the project’s implementation.
5. Schedule feasibility: The purpose of this evaluation is to determine whether the project can be completed within the specified time frame or not.

If TELOS is used to assess the feasibility of dosage forms developing, it may not be appropriate for evaluating the feasibility of IMDs. Thus, IMDs were selected for feasibility assessment to develop and enhance their suitability for evaluating dosage forms. The feasibility study in this study is adapted from Thomayant et al.’s research. ^15^ By improving and changing as follows:

1. Assessment of medical needs and market demand (medical and market need assessment) by gathering information from medical practice guidelines, epidemiological data, marketing databases, or marketing reports. However, the feasibility assessment of the dosage forms may not be evaluated solely based on medical practice guidelines and epidemiological data. Therefore, it should be developed by considering the needs of physicians and drug developers from the industry.
2. Evaluation of competitive potential and intellectual property landscape (key differentiation’s, competitive landscape, intellectual property), where the product should not have any intellectual property restrictions and have unique characteristics for market competition. This can be achieved by searching for information from patent databases, ongoing clinical trials, and other relevant sources. The market feasibility of SR dosage forms development was assessed by examining its growth rate, market competition from various pharmaceutical groups, and the number of drug registrations in Thailand. The production and import values of SR dosage forms were estimated through a search for relevant marketing reports, as well as an analysis of drug usage data from the Thai FDA between 2011 and 2019. Due to the inability of dosage form assessment to evaluate the viability of individual drugs, as noted by Thomayant et al., the assessment process should consider the broader market based on data provided by the Thai FDA.
3. A scientific feasibility assessment should be conducted, including an evaluation of academic evidence supporting both clinical and non-clinical studies. Assessing dosage forms cannot be done in the same way as evaluating new drugs. Therefore, the process should be enhanced by identifying a list of drugs with properties that can be potentially developed into SR dosage forms.
4. The technological feasibility of development should be evaluated to assess the feasibility of developing a commercial product. In technology assessment, the researcher formulates criteria for evaluating the industry’s preparedness to develop a dosage form, which includes quality control capability and assessing the feasibility of developing SR dosage forms.
5. A regulatory feasibility assessment should be conducted to ensure the study meets the criteria and guidelines accepted by the Thai FDA. This includes assessing the clarity of study channels in Thailand and ensuring compliance with drug regulatory guidelines abroad that are recognized by relevant regulatory agencies. The regulatory feasibility is evaluated by conducting interviews to understand the challenges in the field and why there are no new drugs available for registration.

Financial feasibility, ^13^ the research and development period for sustained release of IMDs extended over 7 to 11 years, surpassing the duration of generic drugs due to intricate dosage forms intricacies and heightened instances of project setbacks. The associated developmental costs ranged between 1.46 and 20.23 million USD.

SR is required by the prescribing physician and the domestic pharmaceutical industry is interested in developing IMDs. This research evaluates the distinct requirements of healthcare providers and pharmaceutical manufacturing sectors within the nation. Additionally, there might be a dearth of user or patient preference data. By incorporating an assessment of drug users, it would be feasible to comprehensively gather all the necessary data concerning SR needs.

The marketing aspect is feasible as the SR covers several treatment groups and if the industry can produce by itself, it can increase access to medicines for patients. The local market is comparatively compact. Given that most of the markets within the nation are hospital-based and possess well-defined procurement procedures, the path towards industrial growth and potential exportation might necessitate initiating a well-defined developmental strategy like that of Korea. Such a strategy would promote production, provide export assistance, and ensure convenient patient access to medications. ^5^ Relying solely on the Compound Annual Growth Rate (CAGR), coverage of SR dosage forms, and the attributes of the local pharmaceutical market, the market feasibility evaluation conducted in this study was adequate to arrive at a conclusive determination regarding market viability.

Science is not a concern because the industry can develop drugs. Scientific feasibility assessments can encompass a wide scope, as they often involve the evaluation of a group of dosage forms. However, for the effective development of a new drug, a comprehensive scientific evaluation of all its properties might be essential.

Part of the technological feasibility found that the industry is ready for technology and analytical if assessing the readiness of technology, it is found that industry readiness level 4 according to IMD-TRLs. The technology being research and development by the domestic pharmaceutical industry for manufacturing SR doesn’t demand further investment. However, it might lack the necessary competitiveness in the global market. Therefore, embracing advanced technology for SR development could unlock the potential for both domestic sales and exports.

Guidelines and process for obtaining drug registration approval from the FDA. There is also an opportunity to improve the registration approval guidelines and process for IMDs Guideline. The suggested approach for development, based on the initial guidelines, ^15^ is to provide comprehensive and well-defined instructions. These instructions should be categorized according to distinct dosage forms, and it could be beneficial to include illustrative examples. This would facilitate a better understanding of applicants seeking registration approval. Therefore, it is proposed to establish a structure or organization that supports knowledge or technical knowledge for the approval of the registration of IMDs. Consider establishing an agency or institution to support the industry and the Thai FDA or as a medium for mutual understanding between the two parties.

Therefore, this paper presents key contributions. Firstly, Thailand possesses the capability to create new dosage forms of IMDs presented in the SR dosage forms through its current technological resources. However, to establish a competitive presence in the worldwide market, Thailand might consider advancing its technological methods. Additionally, the pursuit of SR dosage forms development necessitates a comprehensive assessment of medically and scientifically viable drug categories. This study recommends the incorporation of the proposed drug group into this evaluation process.

## Conclusions

The SR dosage forms shows promise for the development of IMDs; however, the major obstacle remains to obtaining drug registration approval.

## Data Availability

Interested readers can contact the corresponding author for access to English excerpts of the interview transcripts ( nusatati@gmail.com). Figshare: Feasibility Study of Sustained Release Dosage forms for Incrementally Modified Drug by Domestics Pharmaceutical Industry in Thailand (Question guideline and Table analysis), https://doi.org/10.6084/m9.figshare.24162417.v4. ^20^ This project contains the following underlying data:
• Appendix A. (Question guideline) • Appendix B. (Table analysis) • Appendix C. (Interview transcription in original language) Data are available under the terms of the Creative Commons Attribution 4.0 International license (CC-BY 4.0).
