# Supplementary material for: Feasibility study of sustained release dosage forms for incrementally modified drug by domestics pharmaceutical industry in Thailand
Source: F1000Res. 2023 Nov 27;12:1513. [Version 1] doi: 10.12688/f1000research.142745.1 (PMC11541073; doi:10.12688/f1000research.142745.1)
Supplement: Peer review history

# Reviewer report: Reviewer response for version 1

**DOI:** 10.5256/f1000research.156326.r333190

**Published:** 2024-11-06

**Authors:** Domenico Fuoco

**Affiliations:** 1 École Polytechnique Montréal, Montréal, Canada

The authors have been very cleavers in structuring this manuscript under a Research Work and not just as a Review of the Domestic Market in Thailand.

 The data reported are easy to validate and the assumptions are aligned with the information reported by the cited works.

 For future ideas:

 Next step for another article could be a General Analysis of Modified Dosage Forms and their trends in the G20 or even more, to understand the patent protection vs medical claims vs API generation in order to have a classification of pharmaceutical needs of Modified Drug Form. See the citation that I am reporting hereto. Ref : 1

Is the work clearly and accurately presented and does it cite the current literature?

Yes

If applicable, is the statistical analysis and its interpretation appropriate?

Yes

Are all the source data underlying the results available to ensure full reproducibility?

Yes

Is the study design appropriate and is the work technically sound?

Yes

Are the conclusions drawn adequately supported by the results?

Partly

Are sufficient details of methods and analysis provided to allow replication by others?

Yes

Reviewer Expertise:

Drug Development, Drug Discovery, Market Access, Pharmacovigilance, Antibiotics. Regulatory Affairs. Patent Analysis. Psychedelics. Nanoparticles. Delivery Systems. Lead Auditor for Good Manufacturing Practice / ISO.

I confirm that I have read this submission and believe that I have an appropriate level of expertise to confirm that it is of an acceptable scientific standard.

## References

[1] : Classification Framework and Chemical Biology of Tetracycline-Structure-Based Drugs. *Antibiotics (Basel)* .2012;1(1) : 10.3390/antibiotics1010001 1-13 10.3390/antibiotics1010001 27029415 PMC4790241

# Reviewer report: Reviewer response for version 1

**DOI:** 10.5256/f1000research.156326.r294545

**Published:** 2024-08-09

**Authors:** Sagar Salave

**Affiliations:** 1 National Institute of Pharmaceutical Education and Research, Ahmedabad, India

1. What is the difference between generic and new generic drugs? Include in the revised manuscript

 2. The author can revise the abstract more in a scientific way

 3. It is advisable to add a few examples of approved SR formulations in the introduction

 4. Pictorial representation for the designed study would improve the readers understanding.

Is the work clearly and accurately presented and does it cite the current literature?

Yes

If applicable, is the statistical analysis and its interpretation appropriate?

Partly

Are all the source data underlying the results available to ensure full reproducibility?

Yes

Is the study design appropriate and is the work technically sound?

Yes

Are the conclusions drawn adequately supported by the results?

Partly

Are sufficient details of methods and analysis provided to allow replication by others?

No

Reviewer Expertise:

Pre-formulation, dosage form design, pharmaceutical technology, Nanomedicine

I confirm that I have read this submission and believe that I have an appropriate level of expertise to confirm that it is of an acceptable scientific standard.
